# *Etlingera elatior* Flower Aqueous Extract Protects against Oxidative Stress-Induced Nephropathy in a Rat Model of Type 2 Diabetes

**DOI:** 10.1155/2022/2814196

**Published:** 2022-04-23

**Authors:** Liza Noordin, Wan Amir Nizam Wan Ahmad, Nor Asiah Muhamad Nor, Nor Hidayah Abu Bakar, Azizah Ugusman

**Affiliations:** ^1^Department of Physiology, School of Medical Sciences, Universiti Sains Malaysia, Health Campus, Kubang Kerian, Kelantan, Penang 16150, Malaysia; ^2^Biomedicine Programme, School of Health Sciences, Universiti Sains Malaysia, Health Campus, Kubang Kerian, Kelantan, Penang 16150, Malaysia; ^3^Faculty of Health Science, Universiti Sultan Zainal Abidin, Gong Badak Campus, Kuala Terengganu 21300, Malaysia; ^4^Faculty of Medicine, Universiti Sultan Zainal Abidin, Medical Campus, Kuala Terengganu 20400, Malaysia; ^5^Department of Physiology, Faculty of Medicine, Universiti Kebangsaan Malaysia, Jalan Yaacob Latif, Cheras, Kuala Lumpur 56000, Malaysia

## Abstract

Diabetes mellitus (DM) is a known systemic disease with increasing global prevalence and multi-organ complications including diabetic nephropathy (DN). The trend of using medicinal plants in the management of DM is increasing exponentially. *Etlingera elatior* is a medicinal plant that contains chemicals and antioxidants that delay the oxidation process. However, available data focusing on its use on DN are inconsistent and scarce. This study aims to investigate the antidiabetic and nephroprotective effects of *E. elatior* flower aqueous extract (EEAE) in a type 2 DM rat (T2DR) model. The T2DR model was developed using a combination of a high-fat diet (HFD) and a low dose of streptozotocin (STZ) at 35 mg/kg. Thirty-two Sprague Dawley male rats were randomly divided into four groups (*n* = 8): (1) control (normal rat), (2) T2DR (untreated-type 2 diabetic rat), (3) Met (250 mg/kg metformin-treated T2DR), and (4) EEAE (1000 mg/kg EEAE-treated T2DR). All treatments were administered orally for 6 weeks. EEAE significantly reduced fasting blood glucose (FBG), microalbuminuria, serum creatinine, and serum blood urea nitrogen. EEAE also reduced malondialdehyde (MDA) and enhanced the levels of antioxidant markers—superoxide dismutase (SOD), catalase (CAT), glutathione (GSH), and total antioxidant capacity (T-AOC). The inflammatory marker (interleukin (IL)-6) and fibrosis markers (transforming growth factor-beta (TGF-*β*), and connective tissue growth factor (CTGF)) were significantly decreased in the EEAE-treated group. The T2DR group developed DN, which was characterized by segmental sclerosis of the glomeruli associated with focal tubular atrophy and interstitial fibrosis. Interestingly, the histology of kidney tissue in the EEAE group was preserved. This effect was similar to that of the control drug metformin. In summary, the antidiabetic and nephroprotective effects might be related to the antioxidant properties and anti-inflammatory effects of the EEAE. The antidiabetic activity could be due to the presence of the active compound cyanidin-3-O-glycosides, which is an anthocyanin antioxidant, that is present in the EEAE. *E. elatior* has the potential to be developed as a natural source of antioxidants that can be used for the prevention or even the treatment of DM. These findings could lead to future research into the therapeutic use of *E. elatior* in alleviating the progression of DM and thus preventing nephropathy.

## 1. Introduction

Epidemiological studies show that the global prevalence of DM has grown, affecting 463 million people, and is estimated to reach over 700 million by 2045 [1]. In Southeast Asia, the prevalence of DM is expected to rise from 11.3% in 2019 to 12.2% in 2030 and further increased to 12.6% in 2045 [2]. The American Diabetes Association (ADA) has classified DM into four categories: type 1 DM (T1DM), type 2 DM (T2DM), specific types of DM, and gestational DM [3]. T2DM is the most common type of DM that contributes more than 90% of all DM cases [4]. It is characterized by chronic hyperglycemia and an inadequate response to circulatory insulin by peripheral tissues, which results in insulin resistance [5]. Complications of DM lead to considerable morbidity and mortality rates, which increase the burden to the healthcare system. DN is one of the most frequent complications in people who have DM. DN is typically defined by macroalbuminuria (>300 mg protein in a 24-hour urine collection) or microalbuminuria (30–300 mg protein in a 24-hour urine collection) with impaired renal function [6]. It was reported that DN is the leading cause of chronic kidney disease worldwide and it develops in about 40% of patients who are diabetic [7]. DN is triggered by various factors including metabolic abnormalities, oxidative stress, fibrosis, inﬂammation, and the activation of the renin-angiotensin system [8]. Hyperglycemia and hypertension have been reported as the most prominent established risk factors for DN [7].

The development and complications of DM have been linked to oxidative stress [9]. Oxidative stress refers to an imbalance between free radical production and the antioxidant system. Hyperglycemia produces reactive oxygen species (ROS) that are responsible for the development of oxidative stress and contributes to the impairment of insulin action and insulin secretion [10]. Multiple factors, including inflammation, mitochondrial dysfunction, and beta-cell malfunction, have been related to the pathophysiology of insulin resistance in DM [5]. A diminished antioxidant enzyme such as SOD, CAT, and glutathione peroxidase (GPx) was demonstrated in patients with DM, which increases oxidative stress [11]. Therefore, seeking an eﬀective therapeutic method is important through supplementation compounds that contain antioxidants. Medicinal plants are a good source of natural medicinal products that contain secondary metabolites with a variety of pharmacological activities. They are known to have chemicals and antioxidants that can delay or inhibit the oxidation process by stabilizing free radicals [12]. Therefore, new strategies that utilize medicinal plants are required to delay the development and the progression of DM. The use of herbal phytoconstituents has been proposed to control blood glucose levels in diabetes by stimulating insulin secretion by pancreatic islets [13] and restoring pancreatic *ß*-cells [14], which are responsible for maintaining *ß*-cell performance.

*E. elatior* is one of the medicinal plants that are widely cultivated throughout Southeast Asia. It belongs to the ginger family, Zingiberaceae, and is commonly known as torch ginger. It is known as “Kantan” in Malaysia and has been used as a fresh vegetable and essential ingredient in Malaysian cuisine [15]. *E. elatior* has traditionally been used as an alternative source of medicine for diabetes and hypertension due to its pharmaceutical properties [16]. We recently demonstrated that EEAE has an antioxidant activity due to a high concentration of secondary metabolites including total phenolic and total flavonoids [17]. This plant has antimicrobial, antioxidant, anticancer, antidiabetic, anti-inflammation, and anti-aging properties [17–20]. Based on the previous studies, *E. elatior* extract was chosen as a potential therapeutic agent in DM. This experiment aims to evaluate the therapeutic eﬀect of *E. elatior* on T2DR and its possible protective effects on DN.

## 2. Materials and Methods

### 2.1. Chemicals

Ghee was purchased from Crispo, Crispo-Tato (*M*) Sdn. Bhd., Kuala Lumpur, Malaysia. Calcium and vitamin D3 were purchased from Eurobio Sdn. Bhd. (Victoria, Australia). Sodium pentobarbitone was purchased from Alfasan Woerden. STZ was purchased from Sigma-Aldrich, Germany, and used to induce experimental diabetes. SOD (Catalog No: E-BC-K020-M), CAT (Catalog No: E-BC-K031-M), GSH (Catalog No: E-BC-K030-M), T-AOC (Catalog No: E-BC-K219-M), MDA (Catalog No: E-EL-0060), IL-6 (Catalog No: E-EL-0060), TGF-*β* (Catalog No: E-EL-0162), and CTGF (Catalog No: E-EL-R0259) kits were purchased from Elabscience, China. Other chemicals were of the highest analytical grade available from local suppliers.

### 2.2. Animals

Thirty-two male Sprague Dawley rats aged 8–10 weeks, weighing 200–220 g, were purchased from Animal Research and Service Centre (ARASC), Universiti Sains Malaysia, Malaysia. The rats were adapted in a controlled environment at the temperature of 23 ± 1°C with a 12-hour light-dark cycle before the experiment and were allowed *ad libitum* access to a standard diet and drinking water. Experimental groups were as follows (*n* = 8/group): (1) control group, (2) T2DR group, (3) Met group, and (4) EEAE group. Group 1 was kept on a standard diet, whereas other groups were induced to be diabetic. The rats of Groups 3 and 4 were given 250 mg/kg Metformin and 1000 mg/kg EEAE orally on daily basis for six weeks, respectively. It was previously shown that the concentration of 1000 mg/kg EEAE possesses antihyperglycemic, antihyperlipidemic, and hepatoprotective effects in TD2R [17]. Hence, this concentration was chosen in this study. All experimental procedures were approved by the Institutional Animal Care and Use Committee (IACUC), USM (Ref: USM/ISCUC/2017/9110(886)).

### 2.3. Induction of Type 2 DM

Twenty-four rats were fed a HFD for six weeks to induce obesity. The nutrition compositions for standard diet and HFD followed our previous study, as shown in Table 1 [21]. The standard rat pellet, Altromin pellet, was imported from Germany by Sterling Ascent, Malaysia. For the preparation of HFD, 32 g of ghee (saturated fat from an animal), 300 mg calcium, and 100 IU of vitamin D3 per 100 g of standard rat pellet were mixed well to become dough-like consistency [21]. Obesity was determined when the body mass index (BMI) was greater than 0.68 g/cm^2^ [22]. The BMI was calculated using the following formula: body weight (*g*)/length^2^ (cm^2^). The rats were injected with a single low-dose STZ at 35 mg/kg intraperitoneally [23]. One-week post-STZ induction, FBG was measured, and rats with FBG levels of greater than 11.1 mmol/L were considered diabetic and thus assigned as T2DR [21].

### 2.4. Preparation of EEAE

**Figure 1:**
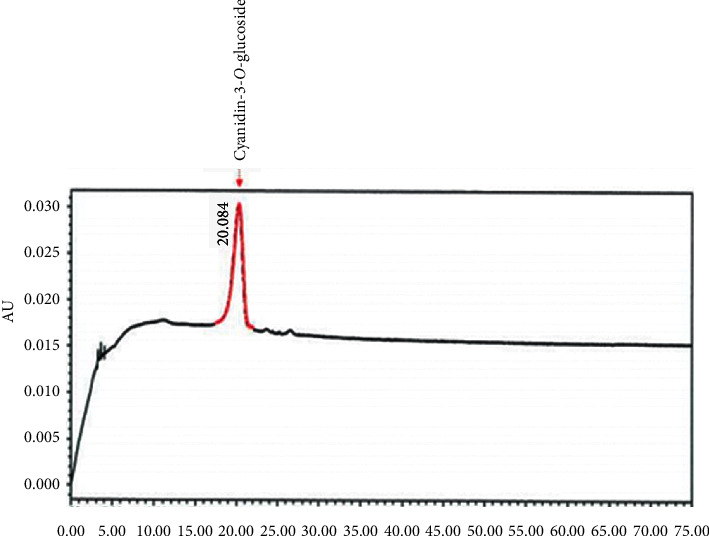
HPLC chromatogram of EEAE with standard reference cyanidin-3-O-glucoside.

*E. elatior* flowers were purchased from a local farmer in Kelantan, Malaysia, and were authenticated at the International Islamic University of Malaysia (IIUM), Pahang, Malaysia, with plant voucher PIIUM 0275. The unopen *E. elatior* flower was selected because it has the highest level of total phenols, flavonoids, and antioxidant properties compared with mature open flowers [24]. The petals were washed, cut into small pieces, and oven-dried at 50°C. The aqueous extract was prepared by an ultrasonic-assisted extraction (UAE) method using a sonicator (SC-221). A 200 g of the sample powder was sonicated three times using 2000 ml, 1800 ml, and 1400 ml of preboiled distilled water at 80°C for 30 minutes each [17]. After that, it was filtered and frozen overnight at −20°C before freeze-drying. The resulting EEAE powder was stored at −20°C until it was further used. Based on the phytochemical analysis, we have demonstrated previously that the EEAE contained phenolic, flavonoid, coumarin, tannin, and quinone [10]. In addition, it has a high total phenolic content [39.06 ± 1.59 mg gallic acid equivalent per gram extract (mg GAE/g)], total flavonoid content [39.00 ± 2.42 mg quercetin equivalent per gram extract (mg QE/g], and total anthocyanin content [54.26 ± 5.34 mg/L] [17]. There was also the presence of cyanidin by using cyanidin-3-O-glycosides standard in high-performance liquid chromatography (HPLC), as shown in Figure 1.

### 2.5. Blood Sampling and Kidney Tissue Isolation

At the end of the experiment, rats were anesthetized with sodium pentobarbitone (60 mg/kg) via intraperitoneal injection. Immediately afterward, blood samples were collected via cardiac puncture. Blood samples for the measurement of oxidative stress parameters were drawn into EDTA-containing tubes and immediately placed on ice. Samples were centrifuged for 10 minutes at 1,000–2,000x g using a refrigerated centrifuge to obtain plasma. Serum samples were assayed for creatinine and blood urea nitrogen (BUN). The kidney was carefully isolated, excised, and rinsed in ice-cold saline to remove red blood cells. For the histopathological study, the kidney was fixed in 10% neutral buffered formalin for 72 hours at room temperature.

### 2.6. Measurement of Biochemical Parameters

FBG levels were measured using the tail-prick method in the dorsal vein using the portable Accu-Check Advantage glucometer (One Touch Ultra). At the end of the experimental period, the rats were placed individually in a metabolic cage and the volume of urine was measured for 24 hours. The urine sample was sent to a local laboratory, and the levels of 24 hours microalbuminuria were measured using the immunoturbidimetric method (Architect, Abbott-Ci8200). Samples for the measurement of microalbuminuria, serum creatinine, and serum blood urea nitrogen were sent to B.P. Clinical Lab Sdn. Bhd., Kota Bharu, Kelantan, Malaysia.

### 2.7. Measurement of Oxidative Stress Markers

The levels of oxidative stress markers such as SOD, CAT, GSH, and T-AOC were measured in the plasma using Biochemical assay kits following the kits' instruction manuals. The activity of SOD was evaluated by the water-soluble tetrazolium salt (WST-1) method. Xanthine oxidase can catalyze the reaction between WST-1 and O_2_^•–^ to generate a water-soluble formazan dye. SOD can inhibit the reaction, and the activity of SOD is negatively correlated with the amount of formazan dye, which can be determined by the colorimetric analysis of WST-1 products. Meanwhile, the determination of CAT activity was evaluated based on hydrogen peroxide decomposition with molybdate through the formation of a yellowish complex, which was detected by colorimetric assay. The intensity of the yellow color corresponds to the amount of CAT bound on the plate. The determination of GSH concentration was done based on the reaction 5,5′-dithiobis-2-nitrobenzoic acid (DTNB) with the sulfhydryl group of GSH to form a yellow complex, which was detected by colorimetric assay. The T-AOC is the cumulative effect of all antioxidants in blood and body fluids. The activity of T-AOC was measured by the 2,2'-azino-bis (3-ethylbenzothiazoline-6-sulfonic acid (ABTS) method. ABTS was oxidized to green ABTS+ by an appropriate oxidant, which could be inhibited with antioxidants. The T-AOC of the sample was determined and calculated by measuring the absorbance of ABTS+. In contrast to other oxidative stress markers, the micro-ELISA plate for the measurement of MDA levels was precoated with an antibody specific to MDA. MDA in the sample or standard competes with a fixed amount of MDA on the solid phase supporter for sites on the biotinylated detection Ab specific to MDA. Excess conjugate and unbound samples or standards were removed from the plate, and horseradish peroxidase (HRP) was added. Each well was filled with a tetramethylbenzidine (TMB) substrate solution, and the stop solution was added to terminate the enzyme-substrate reaction. Finally, the plate was read at a wavelength of 450 nm.

### 2.8. Measurement of IL-6, TGF-B, and CTGF

Kidney tissue was minced into small pieces and rinsed in ice-cold PBS (0.01 M, pH = 7.4) to remove excess blood. Tissue pieces were weighed and homogenized in PBS with the ratio of 1 g tissue weight in 9 mL PBS. Subsequently, the tissue was homogenized with a glass homogenizer kept on ice. The homogenates were then centrifuged for 15 minutes at 5000x g. The supernatant was aliquoted into a small centrifuge tube and stored at −80°C for analysis. Levels of IL-6, TGF-B, and CTGF were measured using ELISA kits following the kits' instruction manuals. The micro-ELISA plate was precoated with an antibody specific to the marker. Briefly, 100 *μ*l of supernatant was added to each of the samples well. After the incubation for 90 minutes at 37°C, a 100 *μ*l biotinylated detection antibody was added to each well. Then, the plate was replaced in the incubator for 60 minutes at 37°C. After a 3-step washing, 100 *μ*l of HRP conjugate was added to each well, and the plate was placed in the incubator again for 30 minutes at 37°C. After the final 5-step washing, 90 *μ*l of substrate reagent was added, with 15 minutes of incubation under darkened conditions. Finally, a 50 *μ*l stop solution was added to the wells and the plate was read at a wavelength of 450 nm.

### 2.9. Histopathological Analysis of Kidney

#### 2.9.1. Hematoxylin and Eosin (H&E) Staining

The paraffin-embedded kidney was sectioned at 3 *μ*m using a rotary microtome (Leica ASP 300S, Germany). The tissue sections were then prepared on the glass slides and placed on a hot plate (HI1220; Leica Microsystems). The tissue sections were then deparaffinized using xylene and rehydrated by immersing them in a series of decreasing concentrations of ethanol (100%, 90%, and 70%). The sections later were stained with H&E to observe the changes in kidney structures.

#### 2.9.2. Periodic Acid-Schiff (PAS) Staining

To demonstrate the kidney's glomerular and tubular basement membrane, the kidney tissue was stained with PAS. The PAS stain highlights the basement membranes. Slides were deparaffinized in a hot plate at 60°C for 1 hour, rehydrated with distilled water, oxidized in 1% periodic acid, and followed by staining with Schiff reagent to stain PAS-positive substance in the tissue. Slides were then washed in running tap water, counterstained with hematoxylin, and turned blue under running tap water. Then, the slides underwent a dehydration process, clearing, and mounting.

#### 2.9.3. Masson's Trichrome (MT) Staining

MT stain was used to demonstrate renal glomerular and tubular fibrosis. Slides were deparaffinized and rehydrated in a series of decreasing concentrations of ethanol and distilled water. Tissue sections were incubated in the Weigert iron hematoxylin for nuclei staining, followed by treatment with a picric acid alcoholic solution. Then, it was incubated in Ponceau acid fuchsin solutions to stain all the acidic tissues. Subsequently, the tissue sections were treated with phosphomolybdic acid and aniline blue. Lastly, slides underwent dehydration process, clearing, and mounting.

#### 2.9.4. Statistical Analysis

Data were analyzed using GraphPad Prism software (version 9) for Windows (GraphPad, San Diego, CA). The results were expressed in mean (standard deviation). A one-way analysis of variance (ANOVA) followed by Tukey's post hoc test was used for statistical analysis. All tests were two-tailed, and a *p*-value of less than 0.05 was considered statistically significant.

## 3. Results

### 3.1. Effects of EEAE on FBG, Microalbuminuria, Serum Creatinine, and BUN

The effects of EEAE on FBG, microalbuminuria, serum creatinine, and BUN on T2DR were evaluated after six weeks. There was a significant reduction of FBG levels in all treatment groups compared with the T2DR group (*p* < 0.001) (Table 2), and the levels were comparable to the control group. Meanwhile, microalbuminuria was significantly lower in the control and treatment groups compared with the T2DR group (*p* < 0.01). Serum creatinine levels were significantly lower in the control and the treatment groups than in the T2DR group (*p* < 0.001). Levels of serum BUN were also significantly lower in the control (*p* < 0.01), metformin-treated T2DR (*p* < 0.001), and EEAE-treated T2DR (*p* < 0.05) groups as compared to the T2DR group.

### 3.2. Effects of EEAE on Oxidative Stress Parameters

Table 3 summarized the results for oxidative stress parameters in the plasma after six weeks of treatment. The MDA levels in the T2DR group were significantly higher when compared with the control (*p* < 0.05), Met (*p* < 0.01), and EEAE (*p* < 0.05) groups. The levels of SOD were significantly lower in the T2DR group when compared with the control (*p* < 0.001), Met (*p* < 0.01), and EEAE (*p* < 0.001) groups. The CAT levels were also significantly lower in the T2DR group when compared with other groups (*p* < 0.001). Meanwhile, the levels of GSH were significantly lower in the T2DR group when compared with the control (*p* < 0.05), Met (*p* < 0.05), and EEAE groups (*p* < 0.01). The levels of T-AOC were significantly lower in the T2DR group when compared with the control (*p* < 0.001), Met (*p* < 0.05), and EEAE (*p* < 0.01) groups.

### 3.3. Effects of EEAE on IL-6, TGF-*β*, and CTGF

**Figure 2:**
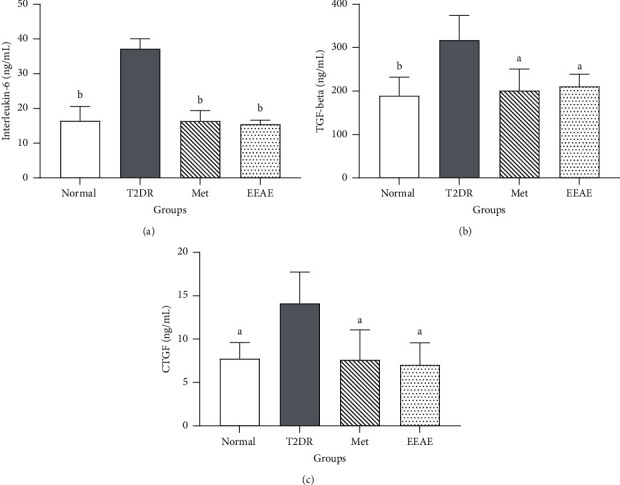
The levels of IL-6 (a), TGF-*β* (b), and CTGF (c) in all groups, *n* = 8 per group. ^*a*^*p* < 0.01, ^*b*^*p* < 0.001 compared to T2DR. T2DR: untreated T2DR, Met: 250 mg/kg metformin-treated T2DR, EEAE: 1000 mg/kg EEAE-treated T2DR, TGF-*β*: transforming growth factor-beta, CTGF: connective tissue growth factor. Statistical analysis was performed using the one-way ANOVA followed by Tukey's post hoc test.

The levels of IL-6, TGF-*β*, and CTGF were measured in the kidney tissue. The levels of inflammatory cytokine (IL-6) were significantly increased in the T2DR group compared with other groups (*p* < 0.01) (Figure 2(a)). The levels of TGF-*β*, a fibrotic marker, were significantly higher in the T2DR group when compared with control (*p* < 0.01), Met (*p* < 0.05), and EEAE (*p* < 0.05) groups (Figure 2(b)). Meanwhile, the levels of CTGF, a fibrotic marker, were also significantly higher in the T2DR group with (*p* < 0.05) as compared to control, Met, and EEAE groups (*p* < 0.05) (Figure 2(c)).

### 3.4. Effects of EEAE on Renal Morphology

#### 3.4.1. H&E Staining

**Figure 3:**
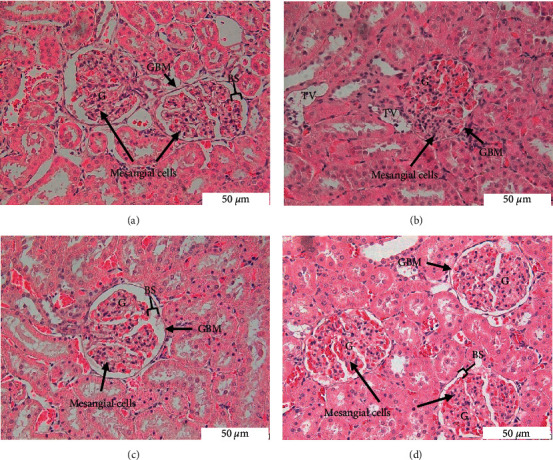
Histological section of kidney stained with H&E at a magnification of 40x. Control (a), T2DR (b), Met (c), and EEAE (d). Glomerulus (G); glomerular basement membrane (GBM); Bowman's space (BS); and vacuolated tubular cells (TV).

The morphological changes in the kidney tissue are shown in Figure 3. In the kidney of the control group, the glomeruli were intact and surrounded by Bowman's capsule (Figure 3(a)). The mesangium that supported the glomerular capillaries has a cluster of three to five nuclei per mesangial area. By contrast, the T2DR kidney exhibited some changes in the glomerular area and kidney tubules (Figure 3(b)). There was a mild thickening of the glomerular basement membrane. In a particular area of the glomerular capillary tuft, a mild segmental increase in the number of mesangial cells was observed. Moreover, a focal area showing vacuolated tubular cells was also seen in this group. In the treatment groups, Met and EEAE showed intact kidney morphology, that is, normal glomeruli and tubules, comparable to the kidney of the control group (Figure 3(c) and 3(d)). However, minimal vacuolated tubular cells remained in the EEAE group (Figure 3(d)).

#### 3.4.2. PAS Staining

**Figure 4:**
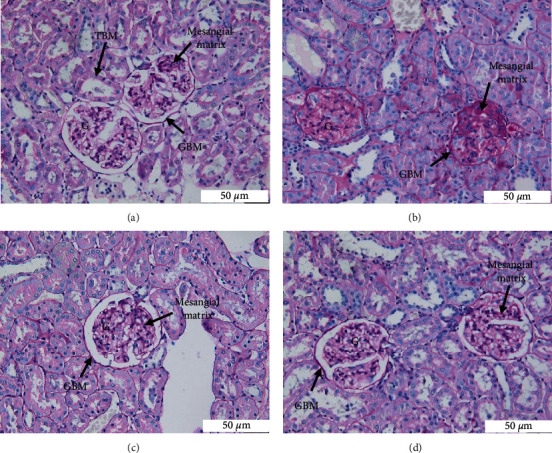
Histological section of kidney stained with Periodic acid-Schiff at 40x magnification. Control (a), T2DR (b), Met (c), and EEAE (d). Glomerulus (G); glomerular basement membrane (GBM); and tubular basement membrane (TBM).

The detailed changes in renal glomerular and tubular tissue demonstrated by the PAS stain are shown in Figure 4. The stain accentuates matrix and basement membrane constituents, protein droplets, and arteriolar hyaline depositions within the kidney tissue. In the control group, the glomeruli exhibited thin and well-defined Bowman's capsule, glomerular capillaries, and tubular basement membrane (Figure 4(a)). By contrast, several glomeruli in the T2DR group demonstrated a segmental area of dense PAS-positive staining associated with segmental areas of mesangial matrix expansion with increased mesangial cellularity (Figure 4(b)). In the Met (Figure 4(c)) and EEAE (Figure 4(d)) groups, the glomeruli and tubular architecture were preserved.

#### 3.4.3. MT Staining

**Figure 5:**
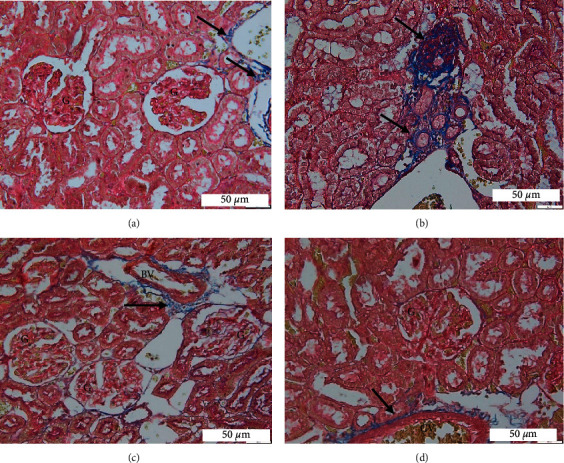
Histological section of kidney stained with Masson's trichrome at a magnification of 40x. Control (a), T2DR (b), Met (c), and EEAE (d). Glomerulus (G); blood vessels (BV); capillary vessels (CV); and collagen deposit (arrow).

The deposition of collagen in renal tissue was illustrated by MT stain. MT stain was used to detect glomerular and interstitial fibrosis. The control group showed no deposition of collagen within the glomeruli and interstitial tissue (Figure 5(a)). By contrast, the T2DR group demonstrated focal fibrosis of the glomeruli associated with a mild increase in the amount of collagen deposition in the interstitial tissue (Figure 5(b)). In the Met (Figure 5(c)) and EEAE (Figure 5(d)) groups, the glomeruli and tubular architecture were preserved, and there was no fibrosis seen within the glomeruli and interstitium.

## 4. Discussion

Evidence suggests that oxidative stress plays a role in the pathogenesis of DM and its complications [25]. Oxidative stress acts as a mediator of insulin resistance and glucose intolerance, contributing to multiple complications including nephropathy, retinopathy, neuropathy, atherosclerosis, and coronary heart disease if left untreated. Antioxidant mechanisms are diminished in diabetic patients that further enhancing oxidative stress [26]. Numerous studies have reported the beneficial effects of antioxidants such as phenolic, flavonoid, and anthocyanin compounds on DM. For example, plant bioactive metabolites such as phenolics, flavonoids, and alkaloids have been shown to have ACE-inhibitory activity and thus have the potential to treat diabetes and its complications [27]. Furthermore, many fruits have been found to contain bioactive compounds with antioxidant properties, such as phenolics, polysaccharides, and vitamin C, which can help restore normal blood glucose levels [28]. The beneficial effects of EEAE on DM and one of its consequences, DN, were investigated in this study. As the composition of bioactive compounds in *E. elatior* is influenced by the maturity stage, location of the cultivated plant, extraction type, and solvent; thus, the phytochemical analysis was carried out earlier.

The effectiveness of EEAE on T2DR was compared to that of metformin, an oral hyperglycemic agent. The findings of this study suggest that *E. elatior* has similar effects to metformin in terms of lowering blood glucose, renal changes, oxidative stress, and inflammation in T2DR. *E. elatior* has antidiabetic activity, which can be attributed to its antioxidant properties and ability to reduce the level of inflammatory markers. Alkaloids, glycosides, flavonoids, saponins, dietary fibers, polysaccharides, glycolipids, and amino acids are potential hypoglycemic agents that play a role in the management of diabetes [29]. The most well-known effects of polyphenols on carbohydrate metabolism are inhibition of *α*-glucosidase and *α*-amylase, the main enzymes responsible for the digestion of dietary carbohydrates into glucose [30, 31]. We have reported recently that EEAE had significant inhibitory activity against *α*-amylase and *α*-glucosidase enzymes [17]. Inhibiting these enzymes causes a delay in carbohydrate digestion, which results in a slower rate of glucose absorption and consequently reduces the postprandial plasma glucose rise [32].

Because most antidiabetic drugs have adverse side effects, DM management remains a challenge [33]. Consuming a variety of natural food sources high in antioxidants is one of the most effective ways to avoid the development of DM. We demonstrated previously that EEAE contains a high concentration of total phenolic and total flavonoids, which exhibit potent antioxidant activity through 1,1-diphenyl-2-picrylhydrazyl (DPPH) and ferric reducing antioxidant power (FRAP) assays [17]. The total phenolic and flavonoid contents of *E. elatior* were found to be high in the leaves, flowers, stems, and rhizome [34]. The flavonoids are classified into various groups, such as anthocyanins, catechins, flavanols, flavones, and flavanones. As a result of the high content of these compounds, the antidiabetic effect of EEAE in the current study is proposed [35, 36]. In the flower of *E. elatior*, four phenolic acids, including gallic acid, tannic acid, chlorogenic acid, and caffeic acid, and five flavonoids, including quercetin, apigenin, kaempferol, luteolin, and myricetin, have been identified [18].

In this study, EEAE shows a high level of cyanidin-3-O-glycosides, using the HPLC method. Cyanidin-3-O-glycoside belongs to the flavonoid family of plant secondary metabolites and specifically to the subgroup of anthocyanins [37]. Anthocyanins are food compounds that are mainly found in dark fruits such as blueberries, black currants, cranberries, and some vegetables such as red cabbage, radish, and eggplant [38]. The beneficial effects of anthocyanin have been reported previously, including anti-inflammatory [39], antimicrobial [40], anticarcinogenic [41], and antidiabetes [42]. Anthocyanins regulate the carbohydrate metabolism in the body by the upregulation of GLUT4 (insulin-regulated glucose transporter) translocation, increased activation of PPAR*γ* (peroxisome proliferator-activated receptor-*γ*) in adipose tissue and skeletal muscles, and increased secretion of adiponectin and leptin [38]. There are six main compounds of anthocyanin that have been identified to have antidiabetic effects including cyanidin, delphinidin, malvidin, pelargonidin, peonidin, and petunidin [42]. In this study, the cyanidin compound was identified in EEAE using the HPLC method. A synergistic effect between anthocyanin and phenolic compounds was responsible for the inhibitory activity of carbohydrate digestive enzymes, antioxidants, and anti-inflammatory activity of pigmented cereals and grains extract [43].

The present study demonstrated that a high-fat diet combined with a single low dose of STZ at 35 mg/kg can result in the development of an animal model of T2DR. The EEAE at 1000 mg/kg has antihyperglycemic effects, which protect the rats from developing DN. After 6 weeks, the EEAE was shown to reduce FBG levels that were comparable to the control drug metformin. In this study, the rats developed DN after six weeks of DM, which was presented by biochemical changes in T2DR, that is, significantly increased microalbuminuria, serum creatinine, and BUN. Microalbuminuria is a biomarker for glomerular and tubular injury [44]. As a result, microalbuminuria is regarded as a predictor of DN [45]. Creatinine and BUN buildup in the body indicated kidney disease that has been reported as a biomarker for kidney degeneration [46, 47]. Diabetes causes renal function impairment due to the accumulation of toxic materials as a result of increased glucose accumulation in the blood [48].

The pathogenesis of DM and its complications are linked to oxidative stress [9, 25]. Hyperglycemia leads to the production of ROS, which causes extensive damage to body cells including the pancreas, liver, and kidneys [49]. We found a decreased level of antioxidant markers including SOD, CAT, GSH, and T-AOC, as well as an increase in MDA level, which results in oxidative stress. Antioxidant enzymes are reduced in diabetic patients, causing oxidative stress to worsen [11]. DM disrupts the lipid profile, making cells more vulnerable to lipid peroxidation, which produces the highly reactive aldehyde, MDA [49]. MDA is a lipid peroxidation product commonly known as a marker of oxidative stress. An elevated MDA level in a diabetic patient suggests that peroxidation injury is involved in the development of diabetes complications. This study indicated that EEAE treatment ameliorated the lipid peroxidation in T2DR and increased the level of antioxidants, similar to that of metformin. Metformin is effective in lowering the level of oxidative stress factors by regulating the cell's antioxidant system [50]. Thus, the current study demonstrated that EEAE could alleviate oxidative stress in T2DR similar to that of metformin.

An increasing number of studies suggested that inflammation, together with oxidative stress and fibrosis, are key links in the progression of DN [8]. The activation of inflammatory pathways is widely recognized as a key mediator in the development and progression of kidney injury [51]. In this study, we discovered increased levels of IL-6, an inflammatory marker, in the serum of T2DR. The thickening of the glomerular basement membrane and mesangial expansion in the kidney is linked to an increase in IL-6 levels [52]. Persistent inflammatory effects may damage glomeruli and cause kidney fibrosis, which is supported by the increased TGF-*β* and CTGF levels in T2DR. Growth factors that are involved in the development of DN include TGF-*β*, vascular endothelial growth factor, platelet-derived growth factor, CTGF, and insulin-like growth factor [53]. TGF-*β* is the primary initiator of fibrosis, which results in tubular cell damage, excess extracellular matrix formation, and inflammatory cell infiltration. These result in impaired kidney function [54]. CTGF has been shown to bind directly to TGF-*β* and causes chronic kidney fibrosis due to their synergistic effect [55, 56]. CTGF expression is regulated by a variety of factors, including angiotensin II, TGF-*β*, hyperglycemia, and cellular stress, all of which contribute to increased kidney fibrosis [57]. Inflammation, together with oxidative stress and fibrosis, are all important factors in the progression of DN [8]. Previous research found that the level of IL-6 in serum was significantly higher in diabetic patients with nephropathy than in diabetic patients without nephropathy, implying that IL-6 may play a role in the pathogenesis of DN [58]. A previous study has found that metformin reduced the production of inflammatory cytokine, IL-6 [59], which supports the present study. Thus, EEAE has also shown an anti-inflammatory effect similar to that of metformin. Besides, EEAE administration significantly decreased renal CTGF and TGF-*β* levels, thus preventing extracellular matrix accumulation and kidney tissue fibrosis.

There were also histopathological changes present in the kidney of T2DR. The H&E staining of kidney tissue revealed morphological changes in the glomerular and tubular areas, which were further confirmed by PAS and MT stains. The stains revealed mesangial expansion and focal segmental sclerosis within the glomerulus of T2DR. DN manifests as excessive mesangial matrix aggregation, thickening of the tubular and glomerular basement membranes, and tubular fibrosis [60, 61]. DN is one of the most common complications in patients with DM that constitutes the primary cause of end-stage renal disease [8]. The impairment of renal function in diabetes is caused by the accumulation of toxic materials, secondary to the increased glucose and fatty acid accumulation in the blood [48]. It is suggested that the imbalance between ROS production and the antioxidant defense system is the main factor responsible for the progression of diabetic kidney diseases [53]. MT stain revealed a mild accumulation of fibrotic tissue in the tubular area. Tubulointerstitial fibrosis occurs following tubular cell hypertrophy, which deteriorates kidney function [62]. The treatment of EEAE restored kidney features in both glomerular and tubular areas. The glucose control in the EEAE group may become the primary prevention of kidney complications through the effects of antioxidant properties of the EEAE. The histopathology of the EEAE group was comparable to the Met group.

## 5. Conclusions

In conclusion, EEAE demonstrated antihyperglycemic and renoprotective effects in T2DR. Oxidative stress has a pivotal role in the pathophysiology of DN. The extract may prevent morphological disruption of the kidney in T2DR by lowering oxidative stress and inflammation. This was proven by the reduction of MDA level and the improvement of the enzymatic antioxidants, T-AOC, SOD, CAT, and GSH in the EEAE group. The antidiabetic activity of EEAE could be due to the presence of active compounds that have antioxidant properties. The phytochemical analysis of EEAE showed a compound of anthocyanin, cyaniding-3-O-glycosides. *E. elatior* has the potential to be developed as a natural source of antioxidants that can be used for the prevention or treatment of DM. Although the exact mechanism has yet to be elucidated, the *E. elatior* diet has the potential to be developed as a therapeutic-based antidiabetic agent.

## Figures and Tables

**Table 1:** Nutritional compositions of standard and high-fat diets.

Compositions (%)	Standard diet (g/100 g)	High-fat diet (HFD) (g/100 g)
Protein	19.2	15.1
Fiber	6.1	18.7
Fat	4.1	31.1
Moisture	11.3	7.0
Ash	6.9	5.2
Carbohydrate	40.8	41.6
Energy (kcal/100 g)	319.8	507.0

**Table 2:** Levels of fasting blood glucose, microalbuminuria, serum creatinine, and urea in experimental groups.

Parameters	Control	T2DR	Met	EEAE
FBG (mmol/L)	4.57 (0.25)^c^	22.43 (1.30)	9.37 (4.26)^c^	9.55 (2.74)^c^
Microalbuminuria (mg/L)	13.33 (0.58)^b^	51.25 (11.18)	18.50 (12.79)^b^	16.75 (7.27)^b^
Serum creatinine (*μ*mol/L)	47.03 (4.03)^c^	69.07 (3.81)	54.67 (4.82)^c^	57.44 (4.56)^c^
Serum BUN (mmol/L)	7.11 (0.65)^b^	9.61 (1.11)	6.46 (1.12)^c^	7.71 (0.99)^a^

Data represent the mean (standard deviation), *n* = 8 per group. ^*a*^*p* < 0.05, ^*b*^*p* < 0.01, ^*c*^*p* < 0.001 compared to T2DR. T2DR: untreated T2DR, Met: metformin-treated T2DR, EEAE: 1000 mg/kg EEAE-treated T2DR, FBG: fasting blood glucose, BUN: blood urea nitrogen. Statistical analysis was performed using the one-way ANOVA followed by Tukey's post hoc test.

**Table 3:** Levels of oxidative stress parameters in experimental groups.

Parameters	Control	T2DR	Met	EEAE
MDA (ng/mL)	187.31 (13.94)^a^	385.72 (27.27)	185.61 (34.30)^b^	217.48 (26.72)^a^
SOD (U/mL)	44.82 (1.81)^c^	32.33 (2.46)	42.39 (0.72)^b^	43.04 (1.01)^c^
CAT (U/mL)	195.65 (27.87)^a^	101.54 (11.42)	185.84 (11.57)^a^	181.26 (18.65)^a^
GSH (*μ*mol/L)	87.26 (15.21)^a^	53.43 (10.47)	87.48 (9.63)^a^	90.89 (18.96)^b^
T-AOC (mmol/L)	3.86 (0.09)^c^	3.65 (0.05)	3.81 (0.07)^a^	3.83 (0.06)^b^

Data represent the mean (standard deviation), *n* = 8 per group. ^*a*^*p* < 0.05, ^*b*^*p* < 0.01, and ^*c*^*p* < 0.001 compared to T2DR. T2DR: untreated T2DR, Met: 250 mg/kg metformin-treated T2DR, EEAE: 1000 mg/kg EEAE-treated T2DR, MDA: malondialdehyde, SOD: superoxide dismutase, CAT: catalase, GSH: glutathione, T-AOC: total antioxidant capacity. Statistical analysis was performed using the one-way ANOVA followed by Tukey's post hoc test.

## Data Availability

The data used to support the findings of this study are available from the corresponding author upon request.
